# Epidemiology of Invasive Fungal Infections in the Mediterranean Area

**DOI:** 10.4084/MJHID.2011.0016

**Published:** 2011-03-31

**Authors:** Ulrike Binder, Cornelia Lass-Flörl

**Affiliations:** Division of Hygiene and Medical Microbiology, Medical University Innsbruck, Austria

## Abstract

Although *Candida* species remain the relevant cause of IFI, other fungi (especially moulds) have become increasingly prevalent. In particular, *Aspergillus* species are the leading cause of mould infections but also *Glomeromycota* (formerly *Zygomycetes*) and *Fusarium* species are increasing in frequency, and are associated with high mortality rates. Many of these emerging infections occur as breakthrough infections in patients treated with new antifungal drugs. The causative pathogens, incidence rate and severity are dependent on the underlying condition, as well as on the geographic location of the patient population. France and Italy show the highest incident rates of *Fusarium* infections in Europe, following the US, where numbers are still increasing. *Scedosporium prolificans*, which primarily is found in soil in Spain and Australia, is most frequently isolated from blood cultures in a Spanish hospital. *Geotrichum capitatum* represents another species predominantly found in Europe with especially high rates in Mediterranean countries. The increasing resistance to antifungal drugs especially of these new emerging pathogens is a severe problem for managing these IFIs.

## Introduction:

Invasive fungal infections (IFIs) are an increasingly important clinical dilemma, engendering high rates of morbidity and mortality, particularly in immunocompromised populations. As a result of growing numbers of patients with a variety of risk factors (e.g. transplantation, chemotherapy, HIV infection, use of corticosteroids or new immunosuppressive agents), the incidence of IFIs has increased substantially in recent years.^1^,^2^,^3^,^4^,^5^,^6^,^7^ For example, aggressive new therapies for transplant recipients and patients with hematologic malignancies have led to more profound immunosuppression of longer duration.^7^

In addition, advances in medical care are extending the survival of critically ill patients, rendering them more vulnerable to IFIs.^1^,^2^,^8^,^9^ The incidence and severity of IFIs as well as the causative pathogens are dependent on various risk factors concerning the patient such as the underlying condition, the state of immunosuppression, but also the geographic location of the patient.^2^,^6^,^10^

A wide variety of pathogens can be associated with IFIs. Historically, *Candida* species have by far been the most common infective organisms among fungi. However, the epidemiology has changed dramatically in recent years: IFIs caused by moulds – predominantly *Aspergillus* species - have increased substantially and newly emerging and rare fungal pathogens such as *Glomeromycota* (e.g. *Rhizopus* and *Mucor* species), hyaline moulds (e.g. *Fusarium* species) and other opportunistic species (e.g. *Scedosporium* species) are increasingly being reported.^7^,^8^,^11^,^12^ This article will predominantly review the most common causatives of IFIs, concentrating on the changing epidemiology of fungal infections and focusing on surveys carried out in the Mediterranean area.

### Yeasts and Yeast-Like Pathogens

#### *Candida* species:

Candida infections are the most frequent cause of IFIs worldwide, with a case rate of 72.8 per 1,000,000 per year^13^ and can result in a wide range of clinical symptoms, from mucocutaneous overgrowth to blood stream infections and metastatic infections.^8^,^14^,^15^ More than 100 *Candida* species have been found to be pathogenic with their frequency varying according to the geographic setting.^16^,^17^,^18^,^19^

The burden of invasive candidiasis remains substantial; after a decline in mortality throughout the early to mid 1990s, mortality rates have leveled off in recent years.^13^ In the United States, *Candida* species are the fourth most common cause of nosocomial blood stream infection.^13^ *Candida (C.) albicans* remains by far the most common species causing invasive candidiasis worldwide (62% in 2003) although the frequency of candidiasis caused by other species including *C. tropicalis*, *C. parapsilosis*, *C. glabrata*, and *C. krusei* has been increasing steadily over the last 10 years.^13^ Two studies in Italy and Spain show the distribution of *Candida* species in the Mediterranean area which was shown to be generally similar to reports from other European countries.^20^,^21^,^22^,^23^

An Italian study^21^ revealed, that *C. albicans* (61 % of all isolates) was followed by *C. parapsilosis*, *C. glabrata* and *C.tropicalis*, which is similar to reports from other European countries, with the only difference that here *C. glabrata* was shown to be the 3^rd^ most common species, while it is the 2^nd^ most common in Switzerland,^24^ the UK^25^ and the US.^26^ Interestingly, in a Spanish study, carried out in Barcelona, *C. glabrata* was shown to be only the 4^th^ most common species, with *C. tropicalis* being the 3^rd^ and *C. parapsilosis* the 2^nd^ most common following *C. albicans*.^27^ The same Spanish study^27^ revealed, that the overall incidence of bloodstream infections caused by *Candida* in Barcelona is lower (4.3 cases per 100 000 population) than in the US (6–10 per 100 000 population).^3^,^28^,^29^ Nevertheless, the number of incidence in Spain correlated well with reports from Northern European countries.^30^,^31^ *Candida* bloodstream infections are in general very high among neonates and infants.^20^,^27^,^32^ With 38.8 cases per 100 000 population the number of incidence in Barcelona/Spain is within the range of numbers obtained from studies in the US.^26^ However, *C. parapsilosis* was the most common species isolated from neonates in Spain (67% of all cases)^20^, whereas in the US *C. albicans* was the most common species and the proportion of *C. parapsilosis* infections was significantly lower (27–45%) than in Spain.^20^,^28^,^29^

Since the 1990s, fluconazole has been widely used for both treatment and prophylaxis of immunosuppressed patients resulting in decreasing rates of *Candida* bloodstream infections worldwide. The downside of this application was that *C. glabrata*, being less susceptible to fluconazole,^3^,^33^ as well as other non-albicans infections are emerging, such as *C. krusei* which is fluconazole resistant.^34^ In a nationwide surveillance study in Spain the frequency of antifungal resistance was determined next to species distribution and incident rates. This study revealed that 7 % of all isolates exhibited decreased susceptibility to fluconazole with a linear correlation to voriconazole resistance. Furthermore, MICs for voriconazole where increased in patients that received fluconazole before, than in those without previous exposure to fluconazole.^35^ Another Spanish study investigated the susceptibility to voriconazole of more than 4000 clinical *Candida* isolates according to EUCAST testing, and revealed that among *C. albicans*, *C. parapsilosis* and *C. tropicalis* resistance to voriconazole was uncommon (with a maximum of 11%), but higher MICs were obtained for *C. glabrata* and *C. krusei*.^36^ The antifungal susceptibility of the *C. parapsilosis*, which recently was found to consist of three different species, namely *C. parapsilosis* sensu strict, *C. metapsilosis* and *C. parapsilosis,* was shown to be low for echinocandins.^37^,^38^ A Portuguese study testing 175 clinical and environmental isolates of the *C. parapsilosis* group showed that the majority (91.4 %) of all isolates are *C. parapsilosis* sensu stricto, and of those most isolates were susceptible to fluconazole. All of the isolates *C. metapsilosis* and *C. parapsilosis* were susceptible to azoles and amphotericinB, while a high number was non-susceptible to echinocandins.^38^ The 10 year ARTEMIS DISK global antifungal surveillance study, where 256 882 isolates of *Candida sp.* were collected from 142 sites in 42 countries and tested against fluconazole, showed that the frequency of azole resistance varied considerably by geographic region.^39^ Higher rates of resistance to both fluconazole and voriconazole were found in isolates from North America. Not only for *C. glabrata* and *C. krusei* decreased susceptibility was shown, but also for *C. guilliermondii*, *C. inconspicua*, *C. rugosa* and others, demonstrating that 13 out of the 31 species found exhibited increased resistance to fluconazole. As described before, cross- resistance between fluconazole and voriconazole is evident and seems to be more pronounced in some species of *Candida* than in others.^39^

#### *Cryptococcus* species:

The genus *Cryptococcus* includes encapsulated yeasts that lack a mycelium.^40^,^41^,^42^ Infection is usually initiated in the pulmonary tract with later possible dissemination, usually to the CNS, causing meningitis.^43^,^44^ Involvement of parenchyma of the brain and meningitis occurs in between 40 and 86% of patients.^44^ Cryptococcosis usually occurs in patients with impaired immunity.^44^ The concern about *Cryptococcus* sp. has dramatically increased as it still remains one of the most common life threatening fungal infections in HIV- patients, where the risk of a *Cryptococcus* infection is between 2.9 – 13.3%. In non-HIV infected individuals, incidence rates of 0.2–0.9% have been reported in the United States.^44^ Patients with AIDS have a much higher risk of infection (2.9–13.3%). In non – AIDS patients, but those with hematologic malignancies, administration of steroids and diabetes mellitus were the most frequent risk factors (6 and 4 out of 17 patients, respectively), as demonstrated in a retrospective study conducted in Italy between 1993 and 2002.^45^

In SOT recipients, an incidence of 2.8% has been reported.^44^ Risk factors for mortality are pre-existing renal failure and liver failure in transplant recipients.^44^

In humans two *Cryptococcus* species can provoke disease: *C. neoformans* and *C. gattii*, which include 5 different serotypes altogether. Two varieties of *C. neoformans* (*C. neoformans var. neoformans* and *C. neoformans var. grubii*) representing serotypes D, A and AD (a hybrid from of both A and D), respectively, have been isolated.^42^,^46^,^47^ *C. gattii* was previously listed as a further variety of *C. neoformans*, but is now known to be a distinct species. *C. gattii* includes serotypes B and C, both commonly seen as true pathogens provoking disease also in immunocompetent persons.^41^,^46^,^48^

*C. neoformans* has a worldwide distribution and has been isolated from a variety of environmental sources, mainly from bird excreta, where the microorganism can survive for a long time due to protection from sun and high temperatures. Its capsule even makes it resistant to natural drying of the vector matter. The distribution of *C. gattii* was thought to be restricted to tropical and subtropical environments, often associated with *Eucalyptus* trees and the koala bear.^49^ Yet, in recent years, an outbreak of *C. gattii* infections has been reported from Vancouver Island/Canada, where more than 66 human cases with at least 4 fatalities have been reported in otherwise healthy persons, all due to infections with serotype B.^50^ In recent years, some other species such as *C. laurentii* and *C. albidus* were isolated from cryptococcosis patients.^51^,^52^

High mortality rates of 30-40%^41^ are mainly due to the difficulty in killing the pathogen. Without treatment, the invasive infection is fatal, which makes rapid diagnosis and treatment inevitable. A combination of amphothericin B and flucytosin, followed by fluconazole maintenance therapy is the therapeutic option in most cases,^53^ although *C. gattii* was found to show resistance to amphotericin B and fluconazole.^54^ Furthermore, a trend of increasing fluconazole resistance of *C. neoformans* isolates from the Asia-Pacific, Africa/Middle East, and Latin America regions but not among isolates from Europe or Northern America has been described in a 10 year antifungal surveillance study.^55^

#### *Trichosporon* species:

Systemic trichosporonosis is a relatively uncommon but frequently fatal opportunistic fungal infection in immunocompromised individuals.^56^ The taxonomy of the yeasts that cause trichosporonosis has been extensively revised.^56^,^57^ It is now widely accepted that the previously named *Trichosporon (T). beigelii* actually consists of six species: *T. asahii*, *T. asteroides*, *T. cutaneum*, *T. inkin*, *T. mucoides*, and *T. ovoides. Geotrichum capitatum*, originally considered a species of *Trichosporon* and now reclassified, is also a common cause of trichosporonosis.^56^ While any immunocompromised patient can develop invasive trichosporonosis, the risk is highest for those with hematologic malignancies.^58^,^59^ Incidence rates of 0.4 and 0.5%, respectively, for infections due to *Trichosporon* sp. and *G. capitatum* have been reported in patients with leukemia.^59^ One of the largest multicenter retrospective studies on invasive trichosporonosis, carried out in Italy,^56^ revealed that acute myeloid leukemia was the most frequent underlying hematologic disease for trichosporonosis. A total of 17 of the 52 patients with hematological malignancies were diagnosed with infections caused by *Trichosporon* sp., while the majority of infections (35 out of 52) was attributed to *G. capitatum*. Furthermore, the study showed that the frequency of *Trichosporon sp.* infections is similar on all continents, while *G. capitatum* is predominantly a European pathogen, with high rates especially countries of the Mediterranean area.^56^ Several *Trichosporon* sp. were shown to be multidrug resistant.^56^ Echinocandins have poor activity against *Trichosporon* sp. as demonstrated by high MICs^58^ and breakthrough cases in immunocompromised patients treated with caspofungin^59^,^60^,^61^ or micafungin^61^ have been reported.

### Moulds

#### *Aspergillus* species:

*Aspergillus* species are opportunistic moulds that can cause both allergic and invasive syndromes.^62^ More than 300 *Aspergillus* species are known today of which only a small number cause opportunistic infections.^62^ The most common species causing aspergillosis is *Aspergillus (A.) fumigatus*, accounting for approximately 90% of *Aspergillus* infections.^63^ Depending on regional distinctions *A. flavus*, *A. nidulans* and *A. terreus* are frequently reported as well, and there is evidence that these non-fumigatus pathogens are increasingly common etiologic agents.^63^,^64^,^65^ There are differences in the clinical presentations produced by these different species.

For example, *A. flavus* produces a disproportionate number of infections in the paranasal sinus, while *A. nidulans* is a common culprit in chronic granulomatous disease.^63^ Although *A. terreus* remains uncommon, infection caused by this pathogen is associated with high mortality rates because of its resistance to amphotericin B.^64^ A study including three European countries, namely Austria, Denmark and Spain, revealed that *A. terreus* seems to be endemic for Tirol, Austria as it was exclusively found in hospital samples from Austria.^66^ In Spain/Madrid *A. niger* was the most isolated non-fumigatus species. Furthermore, it was shown that azole resistance of *Aspergilli* is significantly increasing, especially in the UK (Manchester) and the Netherlands (Nijmegen). The Dutch study, involving almost 2000 *A. fumigatus* isolates collected over a 14-year period in the Netherlands, of which 32 isolates exhibited increased resistance to all azoles tested, showed that 30 of the 32 strains had the same “dominant resistance mechanism”. They all exhibited a single amino acid change in the *cyp51A* gene (encoding the target enzyme cytochrome P450 sterol 14-α-demethylase) and an alteration in the promoter region of this gene. Six isolates out of 317 from other European countries also exhibited resistance to itraconazole. In a study by Pfaller et al. 1789 *Aspergillus* isolates from centers all over the world between 2001 and 2009 were evaluated for their susceptibility to triazoles (voriconazole, posaconazole, itraconazole). For each of the three triazoles tested, decreased susceptibility was observed and varied according to the species. 49 isolates exhibited MICs higher than 4 μg/ml for itraconazole, of which some were shown to be cross resistant to posaconazole and voriconazole.^68^ There exist clinical reports on primary invasive *Aspergillus* infections due to resistant isolates involving various manifestations, e.g. in the lung, the brain, in bones.^69^,^70^,^71^,^72^,^73^ Furthermore, cases have been shown, where itraconazole treatment is lacking clinical efficacy in patients with aspergilloma.^71^,^74^ In Austria the occurrence of azole resistance among clinical *A. fumigatus* is 0% while in Spain it is 2%. Reasons for this increase in resistance are not clear yet, nevertheless there exists some evidence that it is due to excessive use of azoles in agriculture.^75^,^76^

Invasive aspergillosis has remained the predominate cause of invasive mould infections over the last 10–15 years.^77^ Reasons for this include a continued increase in high-risk populations such as solid organ transplant (SOT) and hematopoietic stem cell transplant (HSCT) recipients, HIV-infected individuals, and those receiving intensified chemotherapy regimens.^2^,^63^,^78^,^79^ Invasive aspergillosis is associated with a high rate of mortality, however, there is some evidence that survival rates have increased in recent years among those undergoing HSCT, primarily because of the use of non-myeloablative conditioning regimens, the use of peripheral blood stem cells, prompt diagnosis, and the use of effective antifungal therapy.^6^

An Italian study on invasive aspergillosis in AML-patients (SEIFEM-2008 registry study)^80^ showed that there is a clear downward trend in the aspergillosis-attributable mortality rate. In various consecutive multicenter studies Pagano et al. showed a decrease from 48% (1987–1998), to 38.5% (1999–2003) and 27% (2004–2007).^5^,^80^,^81^,^82^ It is important to note, that according to the latest study,^80^ about two-thirds of the patients developed invasive aspergillosis despite standard antifungal prophylaxis based on fluconazole and itraconazole, which points out that the use of systemic prophylaxis needs to be further discussed. Independently from whatever prophylaxis was applied, *A.fumigatus* was the most causative species of aspergillosis in the Italian study.

#### *Fusarium* species:

*Fusarium* sp. can be found in soil, plants and air. Clinical manifestations are diverse and depend largely on the immune status of the patient. Often, *Fusarium* sp. affects the skin (70–90%), lungs and sinuses (70–80%). Fusariosis is a life-threatening and increasingly important mycosis in immunocompromised hosts.^82^,^83^,^84^ Risk factors for such infections are skin lesions, burns, use of corticosteroids, prolonged neutropenia and hematological malignancy.^12^,^84^,^85^,^86^ *Fusarium* sp. are angiotropic and angioinvasive moulds that produce hemorrhagic infarction and low tissue perfusion, resulting in tissue necrosis.^83^ More than 50 species of *Fusarium* have been identified but only a few are pathogenic in humans.^83^ These include *F. solani* (causes 50% of cases), *F. oxysporum*, *F. moniliforme*, *F. verticillioides, F. dimerum, and F. proliferatum*.^87^

In terms of global occurrence, fusariosis is most common in the United States (50–80% of all cases), followed by France, Italy, and Brazil.^83^,^87^,^88^ In the SEIFEM-2004 survey, *Fusarium* species were responsible for 0.1% of infections, the majority in AML patients (0.3%).^81^ In another Italian study, including 14 haematological centers, *Fusarium* infection was documented in 6 out of 351 patients (1.7%),^88^ with aplastic anaemia and AML as the underlying diseases (3 cases each). While the incidence in Italy remains stable, it increased in some US centers.^84^ Because the clinical presentation of fusariosis may be non-specific, differentiating it from invasive aspergillosis can be challenging.^83^ More than 90% of cases of fusariosis have been reported in neutropenic patients with hematologic malignancies.^88^ Incidence rates of 0.06% (acute leukemia), 0.2% (autologous bone marrow transplant [BMT]), and 1.2% (allogeneic BMT) have been reported.^82^ In patients with hematologic malignancies, persistent neutropenia (hazard ratio [HR] = 5.43) and use of corticosteroids (HR = 2.18) were the most important predictors of mortality. Ideal treatment of fusariosis is still unclear. Azoles and polyenes seem to be most effective. Nevertheless, *Fusarium* sp. exhibit high resistance to antifungal drugs.^81^,^84^,^89^

#### *Scedosporium* species:

*Scedosporium* sp. are ubiquitously distributed worldwide, commonly found in soil, sewage or polluted water. *S. apiospermum* (also known by its teleomorphic name *Pseudoallescheria boydii*) and *S. prolificans* have the greatest impact in human infections.^12^,^90^ These two species differ in their epidemiological niches, morphology and antifungal sensitivity and can cause infections in both immunocompetent and immunosuppressed populations.^85^,^90^ *S. apiospermum* has a worldwide distribution usually in association with water, and is therefore often reported as a cause of pneumonia and disseminated infection in near-drowning victims.^91^ On the other hand, *S. prolificans* is found in soil, mainly in Spain and Australia.^92^ In a Spanish survey, conducted between 1990 and 1999, *S. prolificans* was the most frequent filamentous fungi isolated from blood cultures,^93^ comprising 5.2% of all of the filamentous fungi isolated in the respective hospital (San Sebastion/Spain).^93^

Mycetoma, a disfiguring, but non-life-threatening infection of the skin and subcutaneous tissue, is one type of disease caused by *S. apiospermum*, frequently developed through thorn punctures, wood splinters or preexisting trauma.^11^,^12^,^85^ Pseudallescheriasis or scedosporiosis is mainly found in immunocompromised patients with hematological malignancies or in organ transplant recipients. For 11 % of cases in SOT recipients fungemia with *Scedosporium sp*. was reported.^11^ Interestingly, neutropenia was not a variable in connection with *Scedosporium* infection in SOT patients. Also *S. prolificans* was found to cause deep invasive disseminated infections associated with high mortality rates. Dissemination throughout the body might be easier for this organism due to its ability to produce conidia in tissue.^12^ Both pathogenic species of *Scedosporium* are highly resistant to amphotericin B and echinocandins, with *S. prolificans* being highly resistant to almost all of the currently available antifungal drugs. Voriconazole seems to have the strongest effect on both, *S. apiospermum* and *S. prolificans*, although most data exist from in vitro studies, where MICs for *S. prolificans* are at a level that would not be achieved in human compartiments and not be beneficial for the patient. As an approach, synergistic killing was investigated with a combination of voriconazole and terbinafine, which might be worthwhile to try.^12^,^85^,^94^,^95^,^96^,^97^ Hence, mortality rates have been reported to be as high as 65–75 % for *S. apiospermum* and even higher (85–100%) for *S. prolificans*.

#### *Glomeromycota* (formerly *Zygomycetes*):

Infections with species of the *Glomeromycota* (medically referred to as zygomycosis or mucormycosis) play an increasingly important role in immunocompromised patients. Two orders of the *Glomeromycota* are clinically relevant: *Mucorales* and *Entomophthorales*.^98^,^99^,^100^ Members of the *Mucorales* are distributed worldwide while *Entomophthorales* are generally limited to the tropics and subtropics.^101^,^102^ Species provoking human disease mostly belong to the group of *Mucorales*, which is characterized by a rapidly evolving course, tissue destruction, and invasion of blood vessels.^101^,^103^ The most common species causing mucormycosis are *Rhizopus (R.) arrhizus* (*R. oryzae*), *R. microsporus var. rhizopodiformis*, and *R. pusillus*. Other causative species include *Absidia corymbifera*, *Mucor* species, and *Cunninghamella bertholletiae*.^101^ Mycoses caused by *Entomophthorales* are more indolent and chronically progressive.^101^,^103^

Commonly infections affect the paranasal sinus (39%), the lungs (24%), and the skin (19%) with the primary site of infection depending on the patient population.^7^,^104^ Disseminated disease is reported in approximately one-fourth of patients,^104^ resulting in high mortality rates (96%).^7^ A case-control observational study found that prolonged neutropenie rather than a low neutrophil count is more common in patients with zygomycosis.^104^ Frequent underlying risk factors are diabetes mellitus, particularly enhanced by ketoacidosis, hematological malignances and bone marrow or solid organs transplantation.^85^,^101^,^104^ Diabetes still remains the most common risk factor with 36% to 88% among mucormycosis-cases having diabetes as a predisposing condition.^101^ However, the cases of mucormycosis in patients with hematological malignancies or those who have received hematopoetic stem cell or SOTs is dramatically increasing in the past two decades.^85^ Invasive mucormycosis is now considered to be the 2^nd^ most frequent mould infection in patients with hematological malignances, with reported cumulative incidence ranging from 0.1 – 2.5 % in different series.^104^ An Italian study reports that 45 (11.5%) out of 391 patients with hematological malignancies had infections with a representative of the *Mucorales*.^105^ In France the incidence rate within this patient group increased of 24 % per year from 1997 to 2006.^106^ The so far largest and geographically most diverse study on epidemiology of zygomycosis in Europe, including 15 countries and 230 cases in total, once more pointed out that the most frequent underlying condition for zygomycosis is hematological malignancy (44% of all cases), whereas diabetes is only present in 17 % of all cases.^99^ This is controversial to a study by,^103^ reporting that diabetes account in 36 % of all cases to glomeromycota-infection. One possible explanation for this contrast might be the high increase of immunocompromised hosts in the recent decade.^99^ The presence of available free iron predisposes to zygomycosis.^103^ The application of the iron chelator deferoxamine allows the fungus to utilize deferoxamine-bound iron by recognizing it as a siderophore and enable it to acquire the – for the fungus inevitable – iron via siderophore-specific mechanism/high affinity non-reductive mechanism (sufficient levels of iron increases the ability proliferation and tissue penetration for the fungus). Other chelators (i.e., deferasirox) do not allow iron utilization and may decrease the risk of infection.^107^,^108^ Antifungal prophylaxis with voriconazole also appears to be associated with an increased risk of developing zygomycosis.^85^ For successful eradication of these pathogens a multifactorial treatment strategy is needed. This includes reducing the predisposing factors of the patient, surgical debridement and application of antifungal therapy. Amphotericin B, especially new lipid formulations, is still the agent of choice, and data exist that suggest a combinational therapy with posaconazole as promising.^85^,^109^,^110^,^111^,^112^,^113^

## Conclusion:

With invasive mould infections becoming increasingly important, including those caused by rare, unusual pathogens, the epidemiology of IFIs is shifting in Europe. In some populations mould infections have already overtaken candidiasis, which was once the predominant type of IFIs. Reasons for this shift are multifactorial, but the augmented use of fluconazole as prophylaxis may account, at least in part, for this phenomenon, especially regarding infections with previously rare pathogens that occur as breakthrough infections. The management of IFIs is challenging –complicated by the difficulty in diagnosis and increasing resistance of the pathogens to available antifungal drugs.

## Figures and Tables

**Table 1:** Risk factors and mortality rates of IFIs caused by new emerging pathogens.

**pathogen**	**comments**		**Mortality rate (%)**

	patient population	important facts	

*Cryptococcus gattii*	non-immunosuppressed patients	pulmonary infections outbreaks all due to serotype B expansion of natural habitat resistance to amphotericinB and fluconazole	6 50
*Trichosporon* species	hematological malignancies, neutropenia, SOT	opportunistic infection (part of human skin flora) multi drug resistance high MICs for echinocandins breakthrough infections in caspofungin/micafungin treated populations	65 56
*Fusarium* species	skin leasons and burns, neutropenia, hematological malignancies, contact lenses, HSCT, SOT, corticosteroid treatment	multidrug resistance positive blood culture	70 – 78 89
*Scedosporium* species	near drowning victims (pneumonia and disseminated infection) hematological malignancies, SOT	associated with water (*S. apiospermum*) multidrug resistance infection of skin and subcutaneous tissue (mycetoma) positive blood culture	65 – 100 90
*Glomeromycota*	hematological malignancies diabetes mellitus, neutropenia, SOT, HSCT immunocompetent patients	iron chelator deferoxamine as possible risk factors multifactorial treatment strategy necessary	47 99
